# ROLLIS roll‐out: Pitfalls, errors, lessons learned and recommendations from Australian and New Zealand experience during the randomised controlled trial, implementing a novel localisation method for impalpable malignant breast lesions, radio‐guided occult lesion localisation with iodine‐125 (^125^I) seed (ROLLIS)

**DOI:** 10.1111/1754-9485.13418

**Published:** 2022-05-08

**Authors:** Anita G Bourke, Donna Taylor, Christobel Saunders

**Affiliations:** ^1^ Department of Diagnostic and Interventional Radiology, Breast Centre Sir Charles Gairdner Hospital Perth Western Australia Australia; ^2^ Divison of Surgery, Medical School University of Western Australia Perth Western Australia Australia; ^3^ BreastScreenWA 233 Adelaide Terrace Perth Western Australia Australia; ^4^ Royal Perth Hospital Perth Western Australia Australia; ^5^ Fiona Stanley Hospital Perth Western Australia Australia; ^6^ St John of God Hospital Perth Western Australia Australia

**Keywords:** (125‐I) seed, breast surgery, re‐excision, ROLLIS, WLE

## Abstract

**Introduction:**

Breast cancer surgery aims to excise lesions with clear margins and provide optimal cosmesis with a low re‐excision rates. These aims are aided by accurate lesion localisation and a surgical choice of incision site with minimal removal of healthy tissue. Problems associated with hookwires have led to adoption of non‐wire methods including radioguided occult lesion localisation using iodine‐125 (ROLLIS). This paper outlines the problems encountered and lessons learnt during the largest RCT involving 659 participants, conducted at eight sites (seven Australian, one New Zealand centres) between September 2013 and April 2018.*

**Methods:**

Data, along with substantive comments, regarding each ROLLIS procedure, documenting each step from the seed insertion, ease of operative retrieval, to return of the seed to medical physics, from a shared on‐line secure database and a separate site email survey, were synthesised and categorised.

**Results:**

The Australian and New Zealand ROLLIS RCT experience highlights several important issues. Lessons learned were related to licencing the seed and tracking protocols. A Designated Team Lead, who is a good communicator, ensuring the Tracking Protocols were accurately followed and updated, subspecialty leads and a Co‐ordinator, responsible for training, logbook maintenance and seed ordering, enhanced the success and acceptance of the programme. Addressing radiation issues, fears, education of staff and seed loss was imperative.

**Conclusion:**

The Australian and New Zealand ROLLIS RCT experience highlights the need for adherence to local licencing laws and protocols, appointing a dedicated ROLLIS Designated Team Lead with good communication and a ROLLIS Co‐ordinator. These facilitate the adoption of a successful ROLLIS programme.

## Introduction

**Fig. 1:**
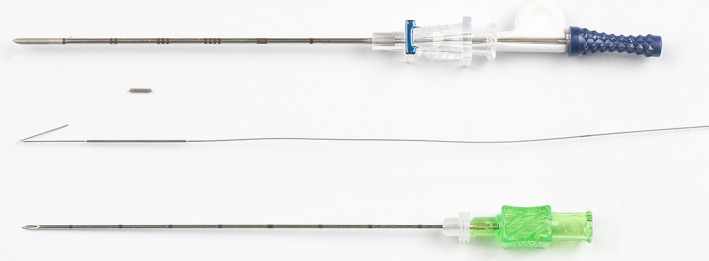
A ROLLIS seed compared to a hookwire. The ROLLIS seed (0.5 mm) and 7 cm deploying needle (blue hub) and spacer (white) are shown (above). A 21G 9 cm hookwire and deploying needle (green hub) are shown (below).

**Fig. 2:**
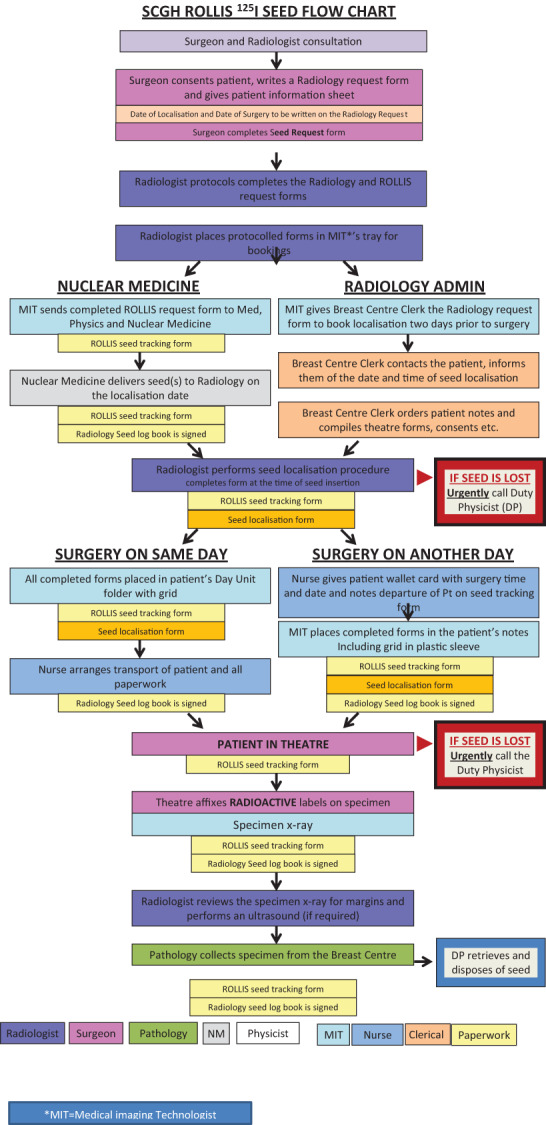
Flow chart. An example of a working flow chart. Each site adopts the protocol to conform to local radiation requirements, accommodate and suit the local practice.

A low dose (<4 Mbq) 4.5 mm × 0.8 mm ^125^I radioactive seed (ROLLIS) (Fig. 1) can be inserted to accurately localise an impalpable breast lesion to guide surgical excision in place of a hookwire (HW) with shorter operating times.^1^, ^2^ The surgeon uses a hand‐held gamma probe to localise the lesion, plan the most appropriate incision, surgically remove the lesion, and in the case of an impalpable breast cancer, a surrounding margin of normal tissue.^3^, ^4^ As the ^125^I seed is a sealed source of radioactivity, there are individual State Radiation Licence requirements that must be fulfilled. A strict seed tracking protocol (see example Fig. 2), which is appropriate for the individual centre, needs to be developed, including a plan to deal with seed loss or damage. A multidisciplinary team approach with good communication is imperative for a successful programme. The pitfalls, errors to be avoided and lessons learned during the Australian and New Zealand ROLLIS RCT are described in this paper, along with suggested solutions.

## Methods

A multicentre RCT, comparing ROLLIS and HWL to guide breast conserving surgery, in 659 female participants with 664 breast lesions which were core biopsy proven either invasive or in situ breast cancer was conducted and completed in April 2018. Eight institutions joined the trial in 2013, seven Australian and one New Zealand site.* Substantial comments, entered in a secure shared on‐line database, incorporated data from each institution regarding each stage of the ROLLIS procedure, from seed insertion, surgical retrieval and seed return to medical physics and an email survey of the Principal Investigators and senior team members at all eight sites, asking for details of any complicated or unexpected ROLLIS experience with tips/hints on how to avoid/overcome the pitfall were reviewed, synthesised and thematically categorised under headings pertinent to each multidisciplinary specialty and these revealed a number of lessons learned during the RCT.

## Results

Issues were identified from a review of the substantive comments in the ROLLIS database and an email survey of all eight sites, yielding 35 comments, which have been incorporated into Lessons and tips related to licencing, the seed and the tracking protocols. Team development included education of current and new staff, equipping staff with a knowledge of radiation issues appointing a Designated Clinical Lead who also had responsibility for ensuring the Tracking Protocols were locally appropriate, functioning, adhered to, updated and setting up Designated Leads in each discipline (Radiology, Surgery, Medical Physics and Pathology) were all recommended useful steps. A ROLLIS Co‐ordinator, who is responsible for training, logbook and seed ordering, enhanced the success and acceptance of the programme.

## Discussion

Most small screen‐detected cancers are impalpable at diagnosis and are treated with WLE for which the most common localisation method in Australia is hookwire localisation.^5^ Problems with wire localisation include scheduling delays and technical challenges.^6^ Complications can occur such as pneumothorax, poor positioning with migration of the wire, either superficially or deep to the lesion and positive margins after wire localisation. Several other localisation methods are available.^7^, ^8^

**Fig. 3:**
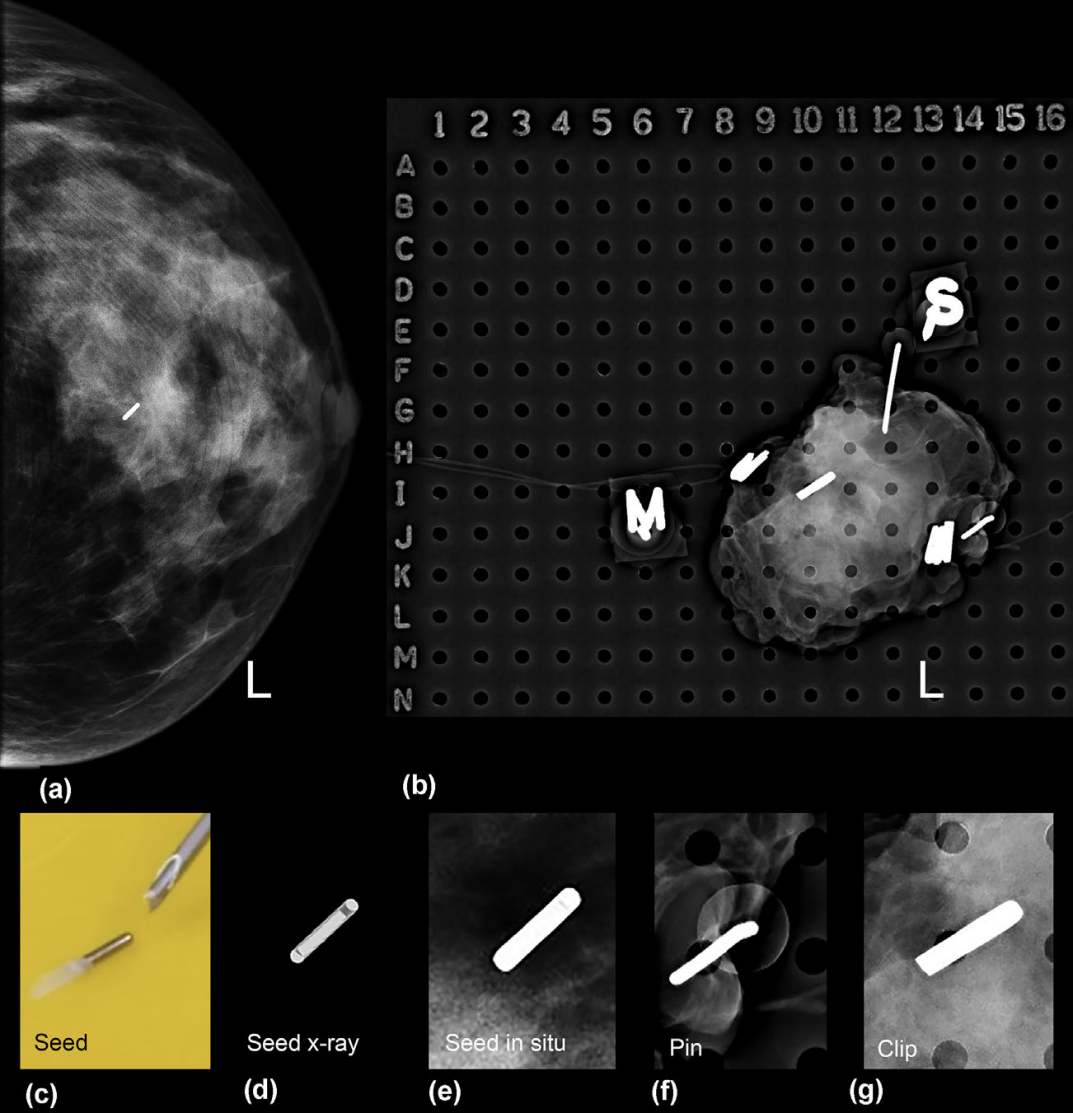
The seed position is confirmed with two mammographic (usually orthogonal) views in all cases. (a) Left (L) cc view showing a radiopaque ROLLIS seed. (b) Specimen radiograph. This was a case of a surgical clip (at co‐ordinates I/10 and image g), which was mistaken for a ROLLIS seed. (c) Shows the wax plug and seed discharged from the needle. (d) Features of the seed include rounded ends and, by hardening the X‐ray window, a small lucency or ‘matchstick’ appearance is seen. The ‘matchstick’ appearance can be more difficult to discern in situ (e) but it distinguishes the seed from pins (f) with a softly opaque halo or a surgical clip (g) with a straight edge on one end. It is important to be familiar with locally used radio‐opaque pins and clips.

Following the successful ROLLIS pilot and extended pilot studies, the use of ^125^I seed received TGA approval in July 2017 for use outside of a clinical trial.^9^, ^10^ ROLLIS has several advantages, including scheduling improvements, as the seed can be inserted up to 8 days prior to the date of surgery (DOS).^11^, ^12^ The advantages of decoupling the same day insertion and surgery offer scheduling flexibility.^11^, ^12^ This temporal decoupling allows greater flexibility in planning of both radiological and surgical lists. In addition, the radiologist chooses the optimal localisation method, ultrasound (US) or stereotactic guidance (SG) and approach, places the seed in the centre of lesion and may place an ‘x’, with indelible ink, on the patient's skin, immediately over the seed, with the patient supine and the ipsilateral arm stretched out to the side. In this study, the radio‐opaque seed position was confirmed mammographically (Fig. 3a) with two, usually orthogonal, views in all cases. The surgeon identifies the ^125^I ‘hot spot’, choses the optimal surgical approach, plans the incision and in real‐time navigates around lesion in 3D, using a gamma probe. Visual and audio signals prompt readjustment, allowing better centring of the lesion within the specimen. This leads to fewer positive margins and equal or lower re‐excision rates.^2^, ^4^, ^11^, ^13^ The technique has been shown to be preferred by women and is easier and faster for the surgeon and radiologist.^14^, ^15^ Better cosmetic results are reported.^16^ The overall complication rate is low.^12^

However, for a successful programme, essential requirements must be addressed. Practical tips from local expert teams, gained during the RCT, include licence issues, setting up a multidisciplinary team, team education, radiation safety and dose, developing local protocols, communication and a plan for avoiding seed loss all of which are discussed below. Factors such as patient and lesion suitability should be considered.

### Licencing

As there is considerable variability across Australia for licencing of sealed sources of radioactivity for a prescriber (either nuclear medicine physician or dual trained radiologist/nuclear medicine physician), an individual user (radiologist/ surgeon/pathologist), teams and premises, it is imperative to check local radiation laws, noting that some states have stringent rules regarding the presence of a physicist when the seed transported and inserted and others require notification if the date of surgery is altered. Team members responsible for radiation safety should contact the Radiation Health Authorities directly in each state and get authorisation for the use of low activity ^125^I seeds at their site. This will require evidence of an adequate staff training programme, including a radiation safety. The Australasian College of Physical Scientists and Engineers in Medicine (ACPEM) have been lobbied for uniform rules for use of low activity seeds for lesion localisation across Australia.

### The ROLLIS team

The sites found a multidisciplinary approach works best with a nominated overall team member, the Designated Clinical Lead (DCL) who takes responsibility for all aspects of the ROLLIS programme.

Disciplinary leaders (DL) in Radiology, Surgery, Theatre and Pathology ensure staff (current and new) training including a radiation quiz and ‘practice run’ is completed prior to observing cases. Mentored sessions with sign off by a senior in that discipline ensure individuals are familiar with and commit to follow the local protocol.

A Programme Co‐ordinator (PC) oversees batch seed ordering and expiry dates, ensures adequate seeds are available, keeps track of seed insertion and removal dates, informs team members of any theatre re‐scheduling changes, manages the mechanism to identify a change of operation day, schedules the use of higher activity seeds later in any delivery batch. This person is responsible for keeping the site‐specific Seed Handling and Tracking Protocols current, training and credentialing of new staff, maintenance of training and records for all staff. Being aware of and ordering seeds according to the supplier's schedule saves costs, with significant additional costs incurred if seeds are ordered outside the delivery schedule.

### Medical physics

The invaluable contribution of the medical physicist is emphasised. The role encompasses maintenance of the gamma probe, keeping a log of seed activities, monitoring the shelf life of the batch to minimise wastage due to device expiry or low activity (<1 MBq). In some centres, seed kits were individually delivered by a medical physicist just before insertion. After the trial, a weekly seed delivery was made, for patients scheduled for that week, in an individually labelled lead envelope and stored in a locked cupboard, in the breast clinic. An ‘on call’ medical physicist is required to attend theatre for assistance with gamma probe issues or a missing seed. Initially, it was useful to have the medical physicist present in theatre.

### Radiology

Occasional seed deployment problems included the seed sticking to the needle tip, which was avoided by rotating the needle 360 degrees about its long axis prior to retrieval. This was easy to check in real time under US guidance. A skin mark was made overlying the lesion with the shoulder joint abducted at 90°.

With stereotactic guidance, ‘Z Zero’ is used as the target deployment position. It was useful to confirm the presence of the seed in the breast with x‐ray, prior to release of compression. Compression release should be slow to avoid seed migration. Standard cranio‐caudal and medio‐lateral orthogonal mammographic views are performed after the seed deployment (Fig. 3a). Early on, superficial seed placement was occasionally noted to cause problems in theatre with the seed falling onto surgical drapes, as in other studies.^12^ The solution was to place the seed on the deep surface of a superficial lesion and inform the surgeon. When bracketing was used for a large lesion or a lesion with linear configuration, it was better to target the medial and lateral aspects or the superior and inferior aspects of a lesion, rather than anterior and posterior (A‐P) bracketing, as it was more difficult for the surgeon to identify 2 signals if they were directly superimposed A‐P. The recommended minimum distance between two seeds is 20 mm.

Seed retrieval in Pathology was aided by accurate radiology reporting of the specimen radiograph (SR), by giving grid co‐ordinates for the seed location (Fig. 3b) or placing a pin at the seed site under x‐ray control. Features of the seed include rounded ends (Fig. 3c), and, by hardening the X‐ray window (Fig. 3d), often a small lucency or ‘matchstick’ appearance is seen and distinguishes it from pins (Fig. 3e,f), mammographic or surgical clips (Fig. 3e,g). In one case, small Liga Clips, being used for the first time, were confused with the presence of the seed (Fig. 3g). Avoiding small Liga clips, using medium‐sized metal clips or other labelling methods, e.g. sutures with metallic side labels, will reduce errors. Informing the radiologists if a new clip is in use is recommended.

The suppliers have given a reassurance (personal communication to Dr Taylor (DT)) that if the manufacturing process changes and the seed has altered in appearance, they will inform users. If a specimen US is performed, the co‐ordinates for seed location should be rechecked on SR. Seed migration has previously been described in 1% of cases.^13^, ^17^, ^18^ and a suboptimal seed position can occur in a small number of cases.^17^, ^18^, ^19^ If the seed was inadvertently misplaced, and the location was suboptimal (>10 mm from the lesion), a ‘backup wire’ was placed in the lesion on the day of surgery and surgeon alerted to use the wire to remove the lesion but also to retrieve the seed. Insertion of a further seed in this situation is not recommended as this may cause confusion. If the seed is placed in the breast prior to the surgical day, there is a requirement for the patient to carry a specially designed wallet card with details including patient identity, the isotope and dose and whom to call in the case of emergency. This is to comply with regulations for seed tracking. There must be a follow‐up system to ensure surgery does occur. The seed tracking form must be placed in the patients notes.

### Surgery and theatre

With regard to Surgical Consent, the patient needs to understand the seed, once inserted, must be removed and returned to medical physics.

Teams found that developing a patient and doctor FAQ sheet, in conjunction with the Breast Care Nurses, was a really useful strategy, especially for new staff who might be asked to gain consent while relatively unfamiliar with the procedure.

To avoid errors in theatre, a discussion should be held at the team ‘time out’ or ‘huddle’ session before the commencement of the theatre list. A nominated nurse is responsible for checking all seed related forms are completed and the specimen is correctly labelled before it leaves the theatre. The seed tracking form must be completed at all stages.

If a sentinel node excision is indicated, it can be performed before or after the lesion excision. However, the surgeon should be aware there can be some downscatter from 99mTc to ^125^I window which is reduced by removing the sentinel node first and then the seed.

To avoid probe malfunction, having both a regular maintenance schedule and a backup probe solved this. If no signal is detected from the seed, check the probe settings. Use 99mTc for the Sentinel Node signal and ^125^I for the seed signal by flicking the switch on the back of the machine.

By checking the cavity, the specimen for ^125^I signal with the gamma probe and the SR, errors in seed loss are avoided. Call the medical physicist early for help if there is any issue. Alteration in the use of surgical clips or staples may affect the SR reporting and so it is important to inform other team members if there are new/substituted items (Fig. 3f).

In addition to a superficial seed falling out onto drapes, not having an immediate SR report from the radiologist can cause confusion.^20^

Seed loss can occur and a seed loss occurred in our trial.^21^ The risk of this happening is minimised with strict protocols. If the seed is not seen, check the breast cavity with the probe. If the seed cannot be found, close theatre access and egress, retain all staff and waste in theatre until the medical physicist evaluates the situation and sets about systematically locating the seed. It is useful to have a Geiger counter in theatre.

### Pathology

The specimen radiograph is used by the pathologist to locate the seed for retrieval either by using co‐ordinates on the grid or by pins in a fresh or fixed specimen.^22^ In one case where a seed was transected in pathology, the protocol was adjusted to use co‐ordinates on an alphanumeric grid to locate the seed, the laboratory was provided with a Geiger counter and new staff were educated to the seed identification method, safe removal and return to medical physicists for delay and decay.

### Protocols, radiation dose and safety

A strict protocol for Departmental Tracking and ‘custody of the seed’ is necessary with an established chain of responsibility, so that each team member knows when and who to call for help and to ensure the protocol is followed. We found regular meetings and exchange of emails helped refine these protocols.

Sealed sources of radioactivity (^125^I) are strictly governed by specific laws even when the radiation dose is low (<4 MBq). The lower energy gamma emission (27 keV) means less tissue penetration but higher absorption.^23^ It has a long half‐life (T 1/2 = 60 days). As an example, a person would need to sit 30 cm from a 3.7 MBq ^125^I seed for 2.7 h per day, 5 days a week, 52 weeks a year (or 140 h @ 1 m from seed for a week) to reach the public radiation limit (1 mSv/year). A method for calculating the radiation dose from an in‐situ seed is available.^24^ A lack of knowledge of the radiation dose can cause confusion.

### Patient/lesion suitability for ROLLIS

^125^I seed localisation is ideal for small lesions, including multiple and/or bilateral lesions. It can be used for localising distortions, masses and calcifications. For larger lesions such as linear/ ductal calcification or lesions with a predominantly elongate growth pattern, it is best to use bracketing wires. For deep lesions in a large breast, a seed with higher specific activity is recommended. ^125^I seeds are not suitable for pregnant women due the low radiation dose.

### Communication

A Designated Lead with a strong focus who addressed concerns early, changed mindsets and led to progressively better patient care. Communication within the relevant departments regarding the patient journey from booking to discharge instilled confidence and collegiality between departments. It was important that staff knew ROLLIS is standard of care.

In conclusion, ROLLIS, using a low‐dose sealed source of ^125^I, has significant advantages over HWL in terms of scheduling, surgical approach and it may reduce re‐excision rates. However, for this programme to be successful, the inter‐disciplinary team needs good communication. The Australian and New Zealand ROLLIS RCT experience highlights the need for adherence to local licencing laws and protocols, appointment a dedicated ROLLIS Designated Clinical Lead and a ROLLIS Co‐ordinator. These facilitate the adoption of a successful ROLLIS programme.

## Ethical approval

Ethics approval was obtained from all hospital sites and the study was undertaken according to the National Statement on Ethical Conduct in Human Research 2007(10).

## Data availability

The data that support the findings of this study are available on request. The data are not publicly available due to confidentiality or ethical restrictions.

## Endnote

Seven Australian (Royal Perth Hospital, Sir Charles Gairdner Hospital, Fiona Stanley Hospital, St John of God Hospital (Perth), Westmead Breast Cancer Institute (Sydney), Monash (Melbourne), Robina (Gold Coast) and one New Zealand site (Waikato, Hamilton).
